# More Accurate Prediction of Metastatic Pancreatic Cancer Patients’ Survival with Prognostic Model Using Both Host Immunity and Tumor Metabolic Activity

**DOI:** 10.1371/journal.pone.0145692

**Published:** 2016-01-04

**Authors:** Younak Choi, Do-Youn Oh, Hyunkyung Park, Tae-Yong Kim, Kyung-Hun Lee, Sae-Won Han, Seock-Ah Im, Tae-You Kim, Yung-Jue Bang

**Affiliations:** 1 Department of Internal Medicine, Seoul National University Hospital, Seoul, Korea; 2 Cancer Research Institute, Seoul National University College of Medicine, Seoul, Korea; INRS, CANADA

## Abstract

**Introduction:**

Neutrophil to lymphocyte ratio (NLR) and standard uptake value (SUV) by ^18^F-FDG PET represent host immunity and tumor metabolic activity, respectively. We investigated NLR and maximum SUV (SUVmax) as prognostic markers in metastatic pancreatic cancer (MPC) patients who receive palliative chemotherapy.

**Methods:**

We reviewed 396 MPC patients receiving palliative chemotherapy. NLR was obtained before and after the first cycle of chemotherapy. In 118 patients with PET prior to chemotherapy, SUVmax was collected. Cut-off values were determined by ROC curve.

**Results:**

In multivariate analysis of all patients, NLR and change in NLR after the first cycle of chemotherapy (ΔNLR) were independent prognostic factors for overall survival (OS). We scored the risk considering NLR and ΔNLR and identified 4 risk groups with different prognosis (risk score 0 vs 1 vs 2 vs 3: OS 9.7 vs 7.9 vs 5.7 vs 2.6 months, HR 1 vs 1.329 vs 2.137 vs 7.915, respectively; *P<*0.001). In PET cohort, NLR and SUVmax were independently prognostic for OS. Prognostication model using both NLR and SUVmax could define 4 risk groups with different OS (risk score 0 vs 1 vs 2 vs 3: OS 11.8 vs 9.8 vs 7.2 vs 4.6 months, HR 1 vs 1.536 vs 2.958 vs 5.336, respectively; *P<*0.001).

**Conclusions:**

NLR and SUVmax as simple parameters of host immunity and metabolic activity of tumor cell, respectively, are independent prognostic factors for OS in MPC patients undergoing palliative chemotherapy.

## Introduction

According to the cancer statistics, pancreatic cancer (PC) is the fifth and fourth leading cause of cancer-related deaths in men and women, respectively [1]. Although rapid progress in the development of targeted therapies has improved overall cancer survival, the prognosis for patients with PC remains disappointing [2].

From the early 21^st^ century, accumulating evidence has revealed that systemic inflammatory response affects tumor growth and metastasis [3, 4]. With an increasing interest in cancer immunity, the mechanisms of immune tolerance to cancer as well as those of anticancer immune response were studied in preclinical animal models and in early clinical trials [5]. The encouraging results of immune checkpoint inhibitors have accelerated the interest in cancer immunity [6, 7].

In recent years, emerging evidence shows that a high neutrophil to lymphocyte ratio (NLR) can be a predictor of poor outcomes in various malignancies, such as colorectal cancer [8], gastric cancer [9], renal cell carcinoma [10], breast cancer [11], and lung cancer [12]. There is also evidence that normalization of NLR after a few cycles of chemotherapy can be used as an early predictor of response to treatment [9, 12], Evidence is accumulating for NLR as an easily accessible tool of immune response and as a prognostic factor in patients with cancer. However, the role of NLR in pancreatic cancer has not been accumulated sufficiently.

Baseline tumor metabolism itself is also an important prognostic factor for cancer outcomes. Although ^18^F-fluorodeoxyglucose (FDG) uptake in positron emission tomography (PET) was shown to quantify tumor metabolism [13], evidence for its usefulness as a prognostic tool is limited in solid tumors including pancreatic cancer.

In this study, we investigated the clinical implication of tumor immunity and tumor metabolism in MPC patients as prognostic parameters. Tumor immunity was evaluated with NLR and change in NLR during chemotherapy, and tumor metabolism was evaluated using a maximum standard uptake value (SUVmax) in ^18^F-FDG PET.

## Materials and Methods

### Study patients

We assessed all consecutive patients with histologically confirmed pancreatic adenocarcinoma, who received palliative chemotherapy at the Seoul National University Hospital between 2003 and 2012. Among the assessed patients, we included only MPC patients, excluding patients with locally advanced pancreatic cancer. We identified the prognostic value of NLR and change in NLR during chemotherapy in all patients and then analysed the prognostic meanings of NLR and SUVmax in patients with ^18^F-FDG PET imaging before palliative chemotherapy (PET cohort).

### Data collection

All relevant clinico-pathological data were retrieved from patient medical records. Laboratory data, including neutrophil and lymphocyte, were obtained within 1 week before the first and second cycle starting dates for first-line chemotherapy. The neutrophils refer to segmented neutrophils and band neutrophils, not including monocytes or myelocytes. The absolute neutrophil count was calculated by the percentage of segmented neutrophils out of the white blood cells. The NLR was determined by the absolute neutrophil count divided by the absolute lymphocyte count. Changes in NLR after one cycle of chemotherapy (ΔNLR) were obtained by subtracting the initial value from the value obtained after one cycle of chemotherapy (cycle 1-cycle 0). Survival time was calculated from the date of diagnosis of MPC to the date of last follow-up or death.

#### Acquisition of ^18^F-FDG PET imaging

PET was usually performed within 1 week prior to starting the first cycle of chemotherapy using integrated PET/CT scanners (Gemini, Philips, Cleveland, OH, USA; Biograph True or mCT40, Siemens, Hoffmann Estates, IL, USA). After fasting for at least 8 hours, ^18^F-FDG (5.18 MBq/kg) was injected, and images were acquired 1 hour later. PET scans were then obtained from the mid-thigh to the skull base, and images were reconstructed using the ordered subset expectation maximum iterative reconstruction algorithm. The SUV was calculated as tissue concentration of radioactivity (kBq/mL) divided by injected dose per weight (kBq/g). To measure the SUVmax of the circular region of interest (ROI), which was defined as the peak SUV in the pixel with the highest count within the ROI, and SUVmax was automatically measured using an analysis software package (Syngo.via, Siemens).

### Statistical analysis

We used the receiver operating characteristic (ROC) curve to determine the best cut-off values for overall survival (OS) with NLR and SUVmax. To compare the between-group differences in demographic and clinical data, continuous variables were converted to categorical variables and examined using the Fisher’s exact test. Median values and mean values of the two groups were compared by logistic regression and independent t-test, respectively. The relationships between continuous variables were assessed by Pearson’s correlation coefficients and Spearman’s *rho*. The median OS was determined using the Kaplan-Meier method. We performed a univariate analysis and then a multivariate analysis using Cox proportional hazard model with forward stepwise selection to evaluate the influence of multiple parameters on survival. Hazard ratios (HR) were reported as relative risk with corresponding 95% confidence intervals (CI). All statistical analyses were performed using the SPSS version 19.0 (IBM Corp. Armonk, NY, USA), and a two-sided *P<*0.05 was considered statistically significant.

#### Ethics

This study was reviewed and approved by the Institutional Review Board of the Seoul National University Hospital (IRB No: H-1307-146-507). All aspects of the study were conducted according to ethical guidelines (Declaration of Helsinki) for biomedical research. Because our study was done with retrospective method, we anonymized patient records/information to be de-identified prior to analysis instead of receiving informed consent from participants for their clinical records to be used in this study.

## Results

### Baseline characteristics

Our study included 396 patients with MPC (Table 1). The median age of all patients was 61 years (range, 20–85 years). Majority of patients (96.0%) received gemcitabine-based chemotherapy including 66 patients (16.7%) with gemcitabine monotherapy, 306 patients (77.3%) with gemcitabine-based doublets, and 24 patients (6.0%) of gemcitabine-based triplets. The remained 16 patients received fluoropyrimidine-based chemotherapy (S1 Table). After the first cycle of chemotherapy, NLR was changed from 2.6 to 1.9 and lymphocyte count, 1522 to 1572, respectively. The cut-off values were as follows: NLR, 2.5 and 4.5 (S1A Fig); ΔNLR, 0 (S1B Fig); and lymphocyte count, 2000. The median OS of all patients was 7.2 months (95% CI, 6.6–7.8 months).

**Table 1:** Baseline characteristics (N = 396).

Characteristics	Range	No. of patients (%)
Gender	Male	252 (63.6)
	Female	144 (36.4)
Age	≥ 60 years	214 (54.0)
	< 60 years	182 (46.0)
ECOG PS	0–1	313 (79.0)
	≥ 2	83 (21.0)
CA19-9	Elevated	319 (80.8)
	Normal	76 (19.2)
Albumin	Decreased	103 (26.0)
	Normal	293 (74.0)
ALP	Elevated	156 (39.4)
	Normal	240 (60.6)
Bilirubin	Elevated	52 (13.1)
	Normal	344 (86.9)
NLR		
Median (range)	2.6 (0.7–37.1)	
Mean ± SD	3.6 ± 3.4	
Range	< 2.5	182 (46.0)
	2.5–4.4	132 (33.0)
	≥ 4.5	82 (20.7)
ΔNLR^a^	< 0 (decreased NLR)	261 (68.5)
	≥ 0 (increased NLR)	120 (31.5)
Lymphocyte	< 2000	297 (75.0)
	≥ 2000	99 (25.0)

ALP, alkaline phosphatase; CA19-9, carbohydrate antigen 19–9; ECOG PS, Eastern Cooperative Oncology Group Performance Status; NLR, neutrophil to lymphocyte ratio; SD, standard deviation.

^*a*^ (NLR after one cycle of chemotherapy)—(initial NLR).

### Analysis of all patients: prognostic value of NLR (N = 396)

**Fig 1:**
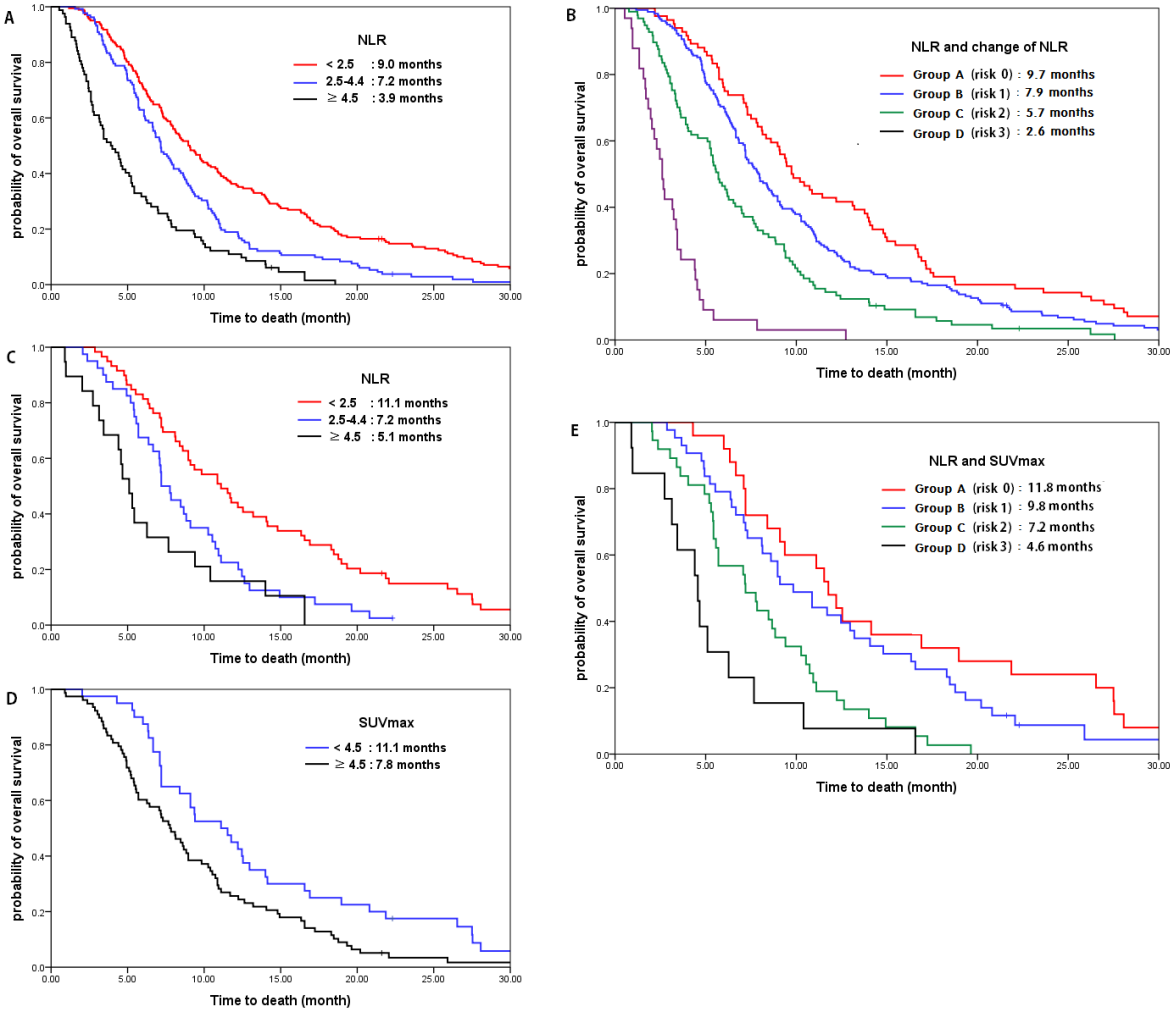
Relationship of NLR and SUVmax with overall survival. (a) Overall survival according to NLR in all patients (N = 396): NLR <2.5: reference; NLR 2.5–4.4: HR 1.659, P<0.001; NLR ≥4.5: HR 2.926 (*P*<0.001). (b) Overall survival according to the risk scores of whole patients (N = 396): Group A (score 0), group B (score 1), group C (score 2), group D (score 3). Total risk score of patients were calculated by addition of each score of NLR (score 0, NLR <2.5; score 1, 2.5≤ NLR <4.5; score 2, NLR ≥4.5) and ΔNLR (score 0: ΔNLR <0; score 1: ΔNLR ≥0). (c) Overall survival according to NLR in PET cohort (N = 118): NLR <2.5: reference; NLR 2.5–4.4: HR 2.113, P = 0.002; NLR ≥4.5: HR 3.500, P<0.001. (d) Overall survival according to SUVmax in PET cohort (N = 118): SUVmax <4.5: reference; SUVmax ≥4.5: HR 1.845, P = 0.004. (e) Overall survival according to risk scores with NLR and SUVmax in PET cohort (N = 118): Group A (score 0), group B (score 1), group C (score 2), group D (score 3). Total risk score of patients were calculated by addition of each score of NLR (score 0, NLR <2.5; score 1, 2.5≤ NLR <4.5; score 2, NLR ≥4.5) and SUVmax (score 0: SUVmax <4.5; score 1: SUVmax ≥4.5).

Univariate analysis of OS identified Eastern Cooperative Group (ECOG) performance status (PS) ≥2 (*P<*0.001), elevated carbohydrate antigen 19–9 (CA19-9) (*P<*0.001), decreased albumin (*P =* 0.006), elevated alkaline phosphatase (ALP) (*P =* 0.002), elevated NLR (*P<*0.001), ΔNLR ≥0 (*P =* 0.049), and lymphocyte count <2000 (*P =* 0.004) as significant factors. Multivariate analysis revealed an increased risk of death in proportion to increases in NLR (NLR <2.5: HR 1; NLR 2.5–4.4: HR 1.659, *P<*0.001; NLR ≥4.5: HR 2.926, *P<*0.001). ΔNLR ≥0 (HR 1.510; *P<*0.001), ECOG PS ≥2 (HR 1.406; *P =* 0.011) and elevated CA19-9 (HR 1.493; *P =* 0.001) were also significant factors (Table 2). As NLR increased, median OS decreased (NLR, <2.5 vs 2.5–4.4 vs ≥4.5; median OS, 9.0 months vs 7.2 months vs 3.9 months; Fig 1a).

**Table 2:** Analysis of factors prognostic for overall survival (N = 396).

Clinical factors	Range	mOS (m)	Univariate analysis			Multivariate analysis		
			HR	95% CI	*P*	HR	95% CI	*P*^b^
Age	≥ 60	7.2	1.052	0.862–1.284	0.616			
	<60	7.4	1					
Gender	Female	7.9	0.883	0.718–1.086	0.240			
	Male	6.8	1					
ECOG PS	≥ 2	5.6	1.665	1.300–2.133	<0.001	1.406	1.082–1.826	0.011
	0–1	7.8	1			1		
CA19-9	Elevated	7.1	1.552	1.251–1.925	<0.001	1.493	1.191–1.872	0.001
	Normal	9.1	1			1		
Albumin	Decreased	6.1	1.380	1.098–1.735	0.006			
	Normal	7.8	1					
ALP	Elevated	5.8	1.373	1.119–1.685	0.002			
	Normal	7.9	1					
Bilirubin	Elevated	6.2	1.126	0.836–1.516	0.435			
	Normal	7.2	1					
NLR					<0.001			<0.001
	<2.5	9.0	1		Reference	1		Referenc
	2.5–4.4	7.2	1.529	1.214–1.925	<0.001	1.659	1.306–2.108	<0.001
	≥4.5	3.9	2.942	2.237–3.869	<0.001	2.926	2.181–3.927	<0.001
ΔNLR^a^	≥ 0	6.1	1.247	1.001–1.553	0.049	1.510	1.204–1.895	<0.001
	< 0	8.0	1			1		
Lymphocyte	< 2000	7.1	1.410	1.119–1.777	0.004			
	≥ 2000	8.6	1					

ALP, alkaline phosphatase; CA19-9, carbohydrate antigen 19–9; CI, confidential; ECOG PS, Eastern Cooperative Oncology Group Performance Status; HR, hazard ratio; mOS, median overall survival; NLR, neutrophil to lymphocyte ratio.

^*a*^ (NLR after one cycle of chemotherapy)—(initial NLR).

^*b*^*P* values were calculated using the Multivariate Cox hazard model adjusted with age, gender, ECOG PS, CA19-9, albumin, ALP, bilirubin, NLR, ΔNLR and lymphocyte by forward stepwise selection.

We made the risk scoring system considering both NLR (score 0, NLR <2.5; score 1, 2.5≤ NLR <4.5; score 2, NLR ≥4.5) and ΔNLR (score 0: ΔNLR <0; score 1: ΔNLR ≥0). By adding 2 risk scores from NLR and ΔNLR, 4 risk groups were identified as follows: group A (risk score 0); group B (risk score 1); group C (risk score 2); group D (risk score 3) (Table 3, Fig 1b). Multivariate analysis showed a gradual increased risk for death with increasing risk scores (group A vs B vs C vs D: 9.7 vs 7.9 vs 5.7 vs 2.6 months; HR 1 vs 1.329 vs 2.137 vs 7.915, respectively; *P<*0.001), CA19-9 (HR 1.494; *P*<0.001) and ECOG PS (HR 1.420; *P* = 0.007).

**Table 3:** Risk groups based on NLR and ΔNLR^a^.

		Clinical factors	N	mOS (m)	HR	95% CI	*P*^b^
Risk group	Risk score						<0.001
A	0	NLR<2.5 &ΔNLR<0	83	9.7	1		reference
B	1	NLR<2.5 &ΔNLR ≥0 or 2.5≤NLR<4.5 &ΔNLR<0	182	7.9	1.329	1.017–1.736	0.037
C	2	2.5≤NLR<4.5&ΔNLR ≥0 or NLR≥4.5 &ΔNLR<0	96	5.7	2.137	1.571–2.906	<0.001
D	3	NLR≥4.5 &ΔNLR ≥0	33	2.6	7.915	5.033–12.445	<0.001

CI, confidence interval; HR, hazard ratio; mOS, median overall survival; NLR, neutrophil to lymphocyte ratio.

^*a*^ (NLR after one cycle of chemotherapy)—(initial NLR).

^*b*^*P* values were calculated using the Multivariate Cox hazard model adjusted with age, gender, ECOG PS, CA19-9, albumin, ALP, bilirubin, and lymphocyte by forward stepwise selection.

### Analysis of the patients in the PET cohort (N = 118)

Among 118 patients in ^18^F-FDG PET cohort, the median OS was 8.6 months (95% CI, 7.4–9.8 months). In this cohort, we obtained a cut-off value of SUVmax of 4.5 through ROC curve to discriminate OS (S2 Fig). There was no correlation between NLR and SUVmax (Pearson *r* = -0.019, *P* = 0.837; S3 Fig) nor any significant difference in the distribution of NLR between high (SUVmax ≥4.5) and low (SUVmax <4.5) metabolism groups (*P =* 0.105) (Table 4).

**Table 4:** Comparison of immune markers between high and low metabolism groups.

	Low metabolism SUVmax<4.5, N = 40	High metabolism SUVmax≥4.5, N = 78	Total, N = 118	*P*
NLR				
Median (range)	2.2 (1.2–19.5)	2.8 (0.8–19.0)	2.5 (0.8–19.5)	0.604^c^
Mean ± SD	3.3 ± 3.5	3.7 ± 3.2	3.5 ± 3.3	0.874^d^
Range				
<2.5	25 (62.5%)	34 (43.6%)	59 (50.0%)	0.105^b^
2.5–4.4	9 (22.5%)	31 (39.7%)	40 (33.9%)	
≥4.5	6 (15.0%)	13 (16.7%)	19 (16.1%)	
Difference of NLR ^a^ ^*(*^ΔNLR)				
< 0	22 (56.4%)	50 (68.5%)	72 (64.3%)	0.220^b^
≥ 0	17 (43.6%)	23 (31.5%)	40 (35.7%)	
Lymphocyte				
< 2000	32 (80.0%)	57 (73.1%)	89 (75.4%)	0.501^b^
≥ 2000	8 (20.0%)	21 (26.9%)	29 (24.6%)	

NLR, neutrophil to lymphocyte ratio; SUVmax, maximum standard uptake value.

^*a*^ (NLR after one cycle of chemotherapy)—(initial NLR)

^*b*^ *P* values were calculated using the Fisher’s exact test

^*c*^ *P* values were calculated using the Logistic regression

^*d*^ *P* values were calculated using the Independent t-test

Multivariate analysis for OS also revealed an increased risk for OS in proportion to NLR (NLR <2.5: 11.1 months, HR 1; NLR 2.5–4.4: 7.2 months, HR 2.113, *P =* 0.002; NLR ≥4.5: 5.1 months, HR 3.500, *P<*0.001; Fig 1c). Patients with high metabolism showed shorter survival than patients with low metabolism (SUVmax <4.5: 11.1 months, HR 1 vs SUVmax ≥4.5: 7.8 months, HR 1.845, *P =* 0.004; Fig 1d) (Table 5).

**Table 5:** Analysis of prognostic factors for overall survival in PET cohort (N = 118).

Clinical factors	Range	mOS (m)	Univariate analysis			Multivariate analysis		
			HR	95% CI	*P*	HR	95% CI	*P*^a^
NLR					0.001			<0.001
	<2.5	11.1	1		Reference	1		Reference
	2.5–4.4	7.2	1.892	1.234–2.902	0.003	2.113	1.330–3.357	0.002
	≥4.5	5.1	3.117	1.810–5.366	<0.001	3.500	1.924–6.366	<0.001
ΔNLR ^b^	≥ 0	7.2	1.145	0.770–1.702	0.503	1.526	1.001–2.328	0.050
	< 0	9.1	1			1		
SUVmax	≥ 4.5	7.8	1.773	1.186–2.651	0.005	1.845	1.209–2.814	0.004
	< 4.5	11.1	1			1		

CI, confidence interval; HR, hazard ratio; mOS, median overall survival; NLR, neutrophil to lymphocyte ratio; PET, positron emission tomography; SUVmax, maximum standard uptake value.

^*a*^*P* values were calculated using the Multivariate Cox hazard model adjusted with age, gender, ECOG PS, CA19-9, albumin, ALP, bilirubin, NLR, ΔNLR, lymphocyte, and SUVmax by forward stepwise selection.

^*b*^ (NLR after one cycle of chemotherapy)—(initial NLR).

We made the risk scoring system considering both NLR (score 0, NLR <2.5; score 1, 2.5≤ NLR <4.5; score 2, NLR ≥4.5) and SUVmax (score 0: SUVmax <4.5; score 1: SUVmax ≥4.5). Using this scoring system, 4 risk groups were identified as follows: group A (risk score 0); group B (risk score 1); group C (risk score 2); group D (risk score 3). Multivariate analysis showed a gradual increased risk for death as risk scores increased (group A vs B vs C vs D: 11.8 vs 9.8 vs 7.2 vs 4.6 months; HR 1 vs 1.536 vs 2.958 vs 5.336, respectively; *P<*0.001; Table 6; Fig 1e).

**Table 6:** Prognostic value of NLR and SUVmax.

Clinical factors			N	mOS (m)	HR	95% CI	*P* ^a^
Risk group	Risk score						<0.001
A	0	NLR<2.5 &SUVmax<4.5	25	11.8	1		reference
B	1	NLR<2.5 &SUVmax≥4.5 or 2.5≤NLR<4.5 &SUVmax<4.5	43	9.8	1.536	0.896–2.630	0.118
C	2	2.5≤NLR<4.5 &SUVmax≥4.5 or NLR≥4.5 &SUVmax<4.5	37	7.2	2.958	1.658–5.279	<0.001
D	3	NLR≥4.5 &SUVmax≥4.5	13	4.6	5.336	2.484–11.461	<0.001

CI, confidence interval; HR, hazard ratio; mOS, median overall survival; NLR, neutrophil to lymphocyte ratio; SUVmax, maximum standard uptake value.

^*a*^*P* values were calculated using the Multivariate Cox hazard model adjusted with age, gender, ECOG PS, CA19-9, albumin, ALP, bilirubin, ΔNLR, and lymphocyte by forward stepwise selection.

## Discussion

In this study, we demonstrated the usefulness of pre-chemotherapy NLR and change of NLR after the first cycle of chemotherapy (ΔNLR) as outcome predictors for MPC patients undergoing palliative chemotherapy. Our results are consistent with previous studies [14, 15]. The new finding in our study was that by scoring system encountered both NLR and ΔNLR, we could identify 4 risk groups of patients with significantly different prognoses (Table 3; Fig 1b).

Cancer-related chronic inflammation promotes angiogenesis and cell proliferation, protects tumors from apoptosis and contributes to metastasis and regional lymph node invasion. This process was known to be initiated with various chemokines that tumor cells secrete and promoted by pro-inflammatory cells, which infiltrate into the tumor microenvironment and make it favorable for cancer progression by the secretion of inflammatory mediators, such as interleukins, tumor necrosis factor-α (TNF-α) and vascular endothelial growth factor (VEGF) [4, 16, 17].

Several recent studies have provided a potential mechanism for increased metastasis in the presence of neutrophilia. The circulating neutrophils could act as a surrogate for the number of tumor-associated neutrophils (TANs), which act as adhesive adapters between circulating tumor cells and the metastatic target [18] and which play an important role in tumor angiogenesis and growth by secreting VEGF and matrix metalloproteinase 9 [19, 20].

Lymphocytes play a substantial role in cell-mediated immunity against tumor cells. CD8+ T-cells are responsible for suppressing tumor growth by inducing cytotoxic T-cell killing, whereas CD4+ T-cells are essential to antitumor immune response. An elevated level of tumor-infiltrating lymphocytes (TILs) is associated with improved outcomes in a variety of cancers [21]. On the other hand, memory T-cells are considered to have a crucial role in carcinogenesis [22]. As a result, lymphopenia is controversial as a poor prognostic factor [23], and its significance was not confirmed in our study.

The most intriguing finding of our study is that host immune response and metabolic activity of the tumor cell itself are independent predictors for outcomes in MPC patients who received palliative chemotherapy. The NLR as a marker of immunity did not correlate with SUVmax as a marker of tumor metabolism (Table 4, S4 Fig). Therefore, we made scoring system consisting of both NLR and SUVmax, which could divide four patient groups with different prognoses (Table 6; Fig 1e). We demonstrated that patients with low NLR and low SUVmax (risk score 0) had longest OS (11.8 months). As the risk score increased to 1, 2 and 3, patient survival was reduced linearly to 0.83 times, 0.61 times and 0.38 times the risk of group A (Spearman *rho* -1.000; S4 Fig). We could establish the prognostic model to more accurately predict patient survival using simple parameters of both host immunity and tumor metabolic activity.

^18^F-FDG PET has already been considered as a predictor of treatment response through more rapid changes in metabolic activity compared to tumor size [24]. The degree of ^18^F-FDG uptake can be semiquantified by SUV, which is an easily measurable and reliable indicator of tumor metabolic activity [25]. However, even though PET was approved to predict final treatment outcomes in lymphoma [26], there remains limited evidence for other malignancies. SUV in PET also could be increased by pancreatitis or peritumoral inflammation, not only by tumor metabolic activity. Recent effort is just limited in methodologic aspect of SUV, not tumor specificity. To obtain a more accurate reflection of the metabolic tumor burden, new PET-based volumetric imaging parameters such as metabolic tumor volume (MTV) and the total lesion glycolysis (TLG) are being attempted for use in various malignancies [27].

Our current study has the limitation of a retrospective approach and needs to be further validated through a prospective study.

In conclusion, pre-treatment NLR and change in NLR after the first cycle of chemotherapy (ΔNLR) could provide predictive information regarding the prognosis of patients with MPC who receive palliative chemotherapy. Furthermore, patient immunity was not correlated with the metabolism of cancer cells themselves. Therefore, consideration of both NLR and SUVmax could provide a more accurate prognosis for patients with MPC. After additional validation studies with a larger cohort, we could potentially apply this easily accessible prognostic model in early decision-making in a clinical setting.

## Supporting Information

S1 Fig ROC curve of NLR (A) and of difference of NLR (B). (TIF)

S2 Fig ROC curve of SUVmax. (TIF)

S3 Fig Correlation between NLR and SUVmax. (TIF)

S4 Fig Linear proportionality of survival to risk score. (TIF)

S1 Table First line chemotherapy (N = 396). (DOC)
