# Supplementary figures and images for: More Accurate Prediction of Metastatic Pancreatic Cancer Patients’ Survival with Prognostic Model Using Both Host Immunity and Tumor Metabolic Activity
Source: PLoS One. 2016 Jan 4;11(1):e0145692. doi: 10.1371/journal.pone.0145692 (PMC4699704; doi:10.1371/journal.pone.0145692)

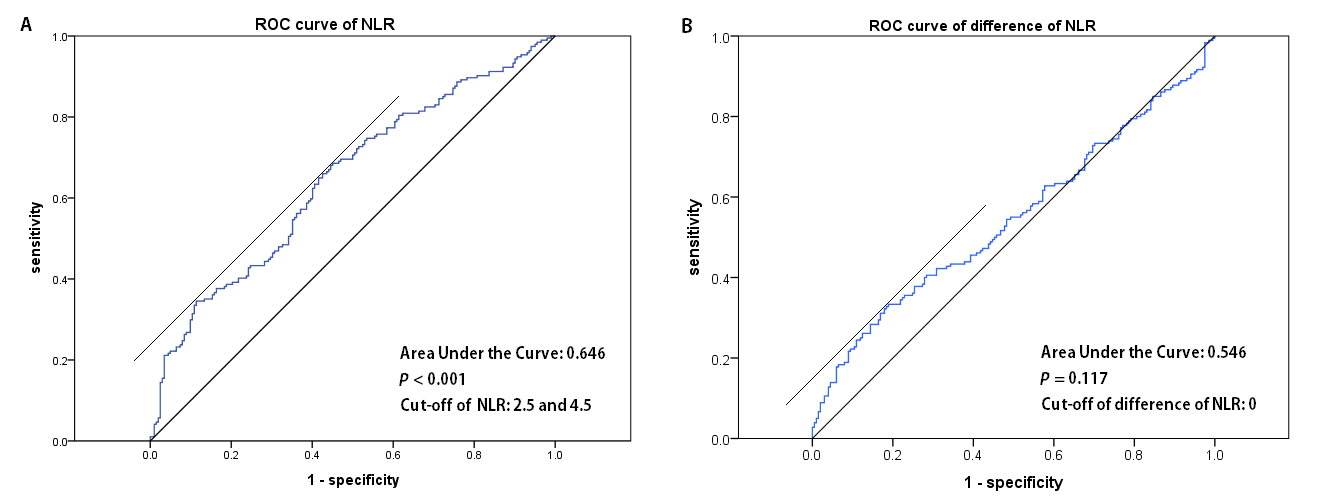

Supplement: S1 Fig — (TIF) [file pone.0145692.s001.tif]

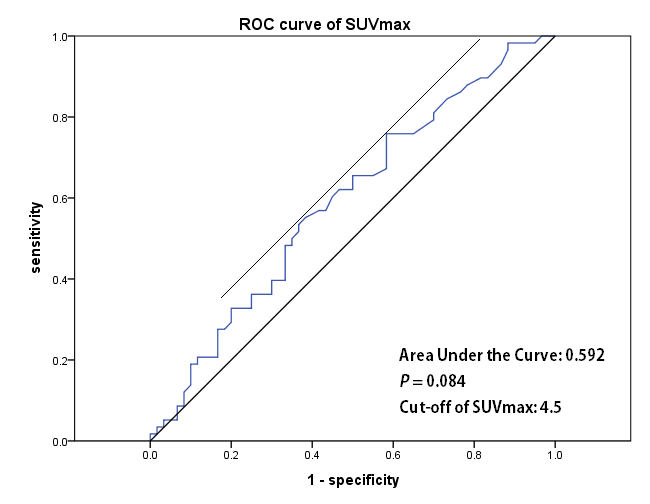

Supplement: S2 Fig — (TIF) [file pone.0145692.s002.tif]

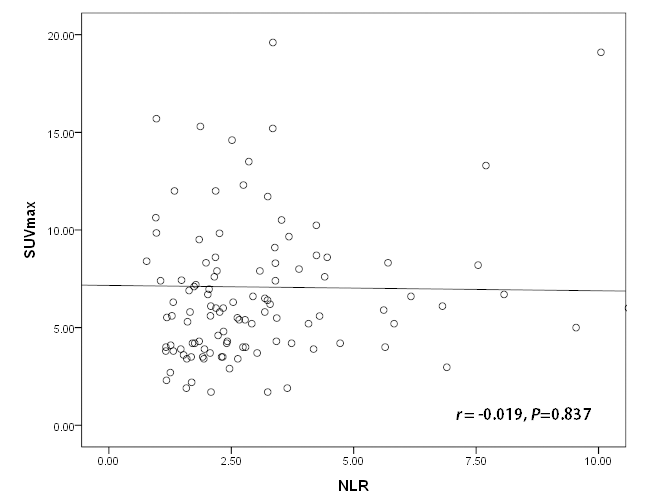

Supplement: S3 Fig — (TIF) [file pone.0145692.s003.tif]

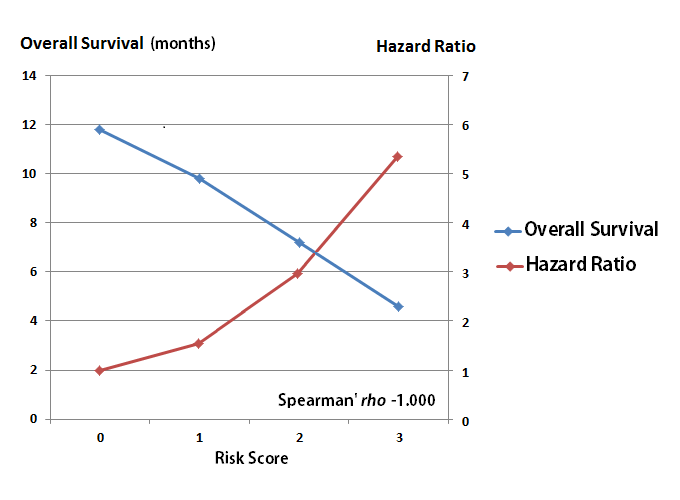

Supplement: S4 Fig — (TIF) [file pone.0145692.s004.tif]
