# Supplementary material for: More Accurate Prediction of Metastatic Pancreatic Cancer Patients’ Survival with Prognostic Model Using Both Host Immunity and Tumor Metabolic Activity
Source: PLoS One. 2016 Jan 4;11(1):e0145692. doi: 10.1371/journal.pone.0145692 (PMC4699704; doi:10.1371/journal.pone.0145692)
Supplement: S1 Table — (DOC) [file pone.0145692.s005.doc]

S1 Table. First line chemotherapy (N=396)

| Gemcitabine-based | N=380 | Fluoropyrimidine-based | N=16 |
| --- | --- | --- | --- |
| gemcitabine | 66 | 5-FU/adriamycin/mitomycin | 9 |
| gemcitabine/cisplatin | 154 | 5-FU/cisplatin | 2 |
| gemcitabine/oxaliplatin | 18 | 5-FU/oxaliplatin | 2 |
| gemcitabine/capecitabine | 71 | TS-1/cisplatin | 1 |
| gemcitabine/erlotinib | 19 | TS-1/oxaliplatin | 1 |
| gemcitabine/docetaxel | 1 | capecitabine | 1 |
| gemcitabine/TS-1 | 10 |  |  |
| gemcitabine/axitinib | 6 |  |  |
| gemcitabine/5-FU | 4 |  |  |
| gemcitabine/MEK inhibitor | 5 |  |  |
| gemcitabine/IGF-1 inhibitor | 2 |  |  |
| gemcitabine/capecitabine/erlotinib | 20 |  |  |
| gemcitabine/cisplatin/erlotinib | 1 |  |  |
| gemcitabine/capecitabine/docetaxel | 2 |  |  |
| gemcitabine/TS-1/erlotinib | 1 |  |  |
